# Mesenchymal Stem Cells Preserve Working Memory in the 3xTg-AD Mouse Model of Alzheimer’s Disease

**DOI:** 10.3390/ijms17020152

**Published:** 2016-01-25

**Authors:** Jiri Ruzicka, Magdalena Kulijewicz-Nawrot, Jose Julio Rodrigez-Arellano, Pavla Jendelova, Eva Sykova

**Affiliations:** 1 Department of Neuroscience, Institute of Experimental Medicine, Academy of Sciences of the Czech Republic, Prague 142 20, Czech Republic; magdalena.kulijewicz@gmail.com (M.K.-N.); j.rodriguez-arellano@ikerbasque.org (J.J.R.-A.); sykova@biomed.cas.cz (E.S.); 2 Department of Neuroscience, 2nd Faculty of Medicine, Charles University, Prague 150 06, Czech Republic; 3 Functional Neuroanatomy Laboratory, Department of Neuroscience, Faculty of Medicine, the University of the Basque Country, 48940 Leioa, Spain

**Keywords:** Alzheimer’s disease, mesenchymal stem cells, working memory, Aβ*56, neurogenesis

## Abstract

The transplantation of stem cells may have a therapeutic effect on the pathogenesis and progression of neurodegenerative disorders. In the present study, we transplanted human mesenchymal stem cells (MSCs) into the lateral ventricle of a triple transgenic mouse model of Alzheimer´s disease (3xTg-AD) at the age of eight months. We evaluated spatial reference and working memory after MSC treatment and the possible underlying mechanisms, such as the influence of transplanted MSCs on neurogenesis in the subventricular zone (SVZ) and the expression levels of a 56 kDa oligomer of amyloid β (Aβ*56), glutamine synthetase (GS) and glutamate transporters (Glutamate aspartate transporter (GLAST) and Glutamate transporter-1 (GLT-1)) in the entorhinal and prefrontal cortices and the hippocampus. At 14 months of age we observed the preservation of working memory in MSC-treated 3xTg-AD mice, suggesting that such preservation might be due to the protective effect of MSCs on GS levels and the considerable downregulation of Aβ*56 levels in the entorhinal cortex. These changes were observed six months after transplantation, accompanied by clusters of proliferating cells in the SVZ. Since the grafted cells did not survive for the whole experimental period, it is likely that the observed effects could have been transiently more pronounced at earlier time points than at six months after cell application.

## 1. Introduction

Alzheimer’s disease (AD) is the most common cause of dementia and accelerates with advancing age. AD is clinically defined as progressive cognitive impairment or memory loss. AD is characterized by three major neuropathological hallmarks: senile plaques composed of *β*-amyloid (A*β*) peptides, intracellular neurofibrillary tangles, and neuronal loss. The loss of neurons and synapses spreads to the hippocampus, entorhinal cortex and frontal cortex, all of which play important roles in reference and working memory [1,2,3,4]. Additionally, microglial cells and astrocytes are highly affected by AD [5]. Stem cells show the potential for trophic support, immunomodulation, neurogenesis and the reduction of plaque deposits via the modulation of microglia activation [6,7,8]. To date, a variety of stem cell types have been used to treat transgenic animal models of familial and sporadic AD. Apart from neural stem cells (NSCs), mesenchymal stem cells (MSCs), which can be isolated from bone marrow [8,9,10], adipose tissue [11], umbilical cord blood [7,12] and other sources, have been used to examine their potential in AD treatment. Some authors have reported that grafted stem cells can decrease amyloidogenesis, increase synaptogenesis and prevent neurons from degenerating in the brain of AD animal models, and due to their ability to differentiate and proliferate in these models, the grafted cells might replace lost neurons in the brains of AD patients [13,14,15]. Cholinergic neurons are often targeted as clinically relevant due to their importance in memory function. However, despite the possibility of NSCs differentiating *in vivo* into a more mature neuronal/glial phenotype, neuronal replacement is still the subject of debate due to the limited appearance of pathological neuronal loss in animal models [16]. However, the delivery of neurotrophic factors from NSCs and MSCs obtained from various sources has shown a positive effect on degenerating neurons. The transplantation of NSCs genetically modified to overexpress neurotrophins led to a significant improvement of memory deficits in AD mouse models via the suppression of apoptosis and the maintenance of functional synaptic contacts [14,17,18,19,20]. Similar to NSCs, MSCs have been shown to have the ability to restore lesioned brain areas by secreting trophic factors such as growth factors and anti-inflammatory cytokines and to facilitate A*β* plaque clearance via microglia [7,8,10,12]. Some reports have shown the increased expression of proteins related to cognitive function and synaptogenesis after stem cell application in comparison with non-treated AD animals [20,21]. MSCs have an advantage for clinical application in that expanded autologous as well as allogenic MSCs are non-immunogenic [22,23,24]. Adequate animal models of AD that mimic human cases and follow human pathogenesis are required to evaluate the effects of cell transplantation. The triple transgenic mouse model of AD (3xTg-AD) generated by LaFerla and colleagues [25] harbors three transgenes of Presenilin 1 (PS1, M146V), tau (P301L) and Amyloid precursor protein (APP, SWE) and manifests the two major pathological features seen in AD patients, namely A*β* plaques and neurofibrillary tangles. Extracellular amyloid plaques are observed in 3xTg-AD animals in the frontal cortex at six months of age and in the hippocampus by 12 months followed by neurofibrillary tangles, leading to a memory deficit measurable in the Morris water maze (MWM) test [1,26,27].

In our study, human MSCs (hMSCs) were transplanted into the lateral ventricle of 3xTg-AD mice, and their effects were examined behaviorally and histologically. Here we report beneficial effects of the implanted hMSCs; these effects were found at 14 months of age, six months post-transplantation.

## 2. Results

### 2.1. Reference and Working Memory

**Figure 1:**
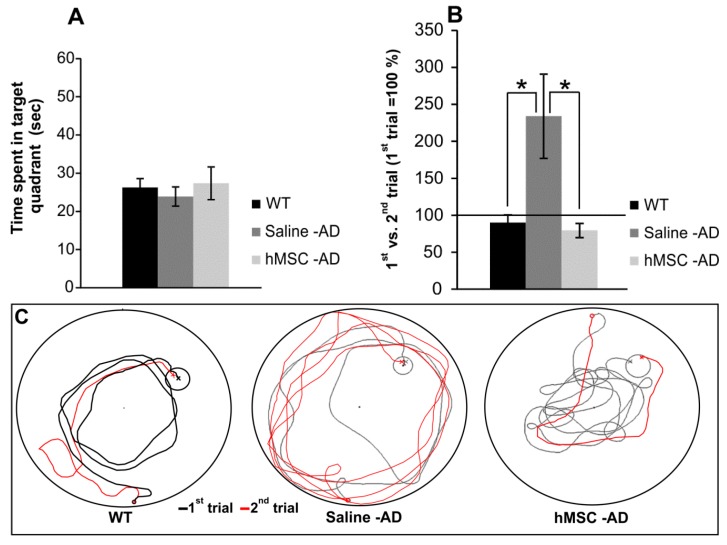
Results from the Morris water maze (MWM) test (probe trial) showing that reference memory was maintained in non-transgenic and transgenic mice at 14 months of age (**A**); In the working memory test, the percentages shown on the y-axis indicate what percent of the time needed to find the hidden platform was saved between the first and second trials; 100% indicates no difference between the two trials (**B**); Illustrative images of mouse trajectories in the MWM during the first and second trials used for working memory assessment (**C**). * *p* < 0.05. WT: wild-type; AD: Alzheimer’s disease; hMSC: human mesenchymal stem cell.

Spatial reference memory and working memory were evaluated at 14 months of age (six months after treatment or without treatment). In parallel with the treated transgenic mice, non-treated non-transgenic mice (C57BL6 strain, which is the strain used to generate 3xTg-AD mice) were used as age-matched controls. Reference memory was tested in the Morris water maze. The latency to reach the submerged platform on the last day of the ten-day training period was 14.7 ± 1.6 s in wild-type (WT) mice, 19.7 ± 3.0 s in saline-treated Tg mice, and 21.5 ± 2.8 s in MSC-transplanted Tg mice; there were no significant differences between the three groups (*p* > 0.05). The probe trial, in which the hidden platform was removed, showed that the time spent in the correct quadrant where the hidden platform had been previously placed during the ten-day training period was 26.2 ± 2.3 s in WT mice, 23.8 ± 2.5 s in saline-treated Tg mice, and 27.3 ± 4.3 s in MSC-transplanted Tg mice. Again, there were no significant differences between the groups (*p* > 0.05). Reference memory was maintained in both non-transgenic and transgenic mice at 14 months of age (Figure 1A) as there were no statistically significant differences in the performance of WT and transgenic mice at 14 months of age and their performance at five months of age (data not shown).

After the reference memory test, working memory was examined in the same Morris water maze tank, but the tank was modified. Working memory was evaluated between the first and second trials on each day of testing. If an animal could remember the location where it found the submerged platform during the first trial, it could then save time in finding the platform during the second trial. If the time needed to find the platform during the second trial is divided by the time needed during the first trial and expressed as a percentage, a value of more than 100% is defined as working memory impairment because the animal was unable to save time in finding the platform between the two successive trials. Approximately 85% of saline-injected Tg mice, 20% of MSC-injected Tg mice and 29% of non-treated WT mice could not save time in finding the platform during the second trial. The percentages are shown in Figure 1B: 89.7% ± 11.0% in non-treated WT mice, 234.0% ± 57.1% in saline-injected Tg mice, and 79.4% ± 9.7% in MSC-injected Tg mice. There were significant differences between WT mice and saline-injected Tg mice and between MSC-injected Tg mice and saline-injected Tg mice (*p* < 0.05). Transgenic mice without MSC treatment lost their working memory, but this impairment was not observed in the transgenic mice that received transplanted MSCs (*p* > 0.05).

### 2.2. Influence of Human Mesenchymal Stem Cells (hMSCs) on Amyloid β (Aβ*56) and Glutamine Synthetase (GS) Levels

**Figure 2:**
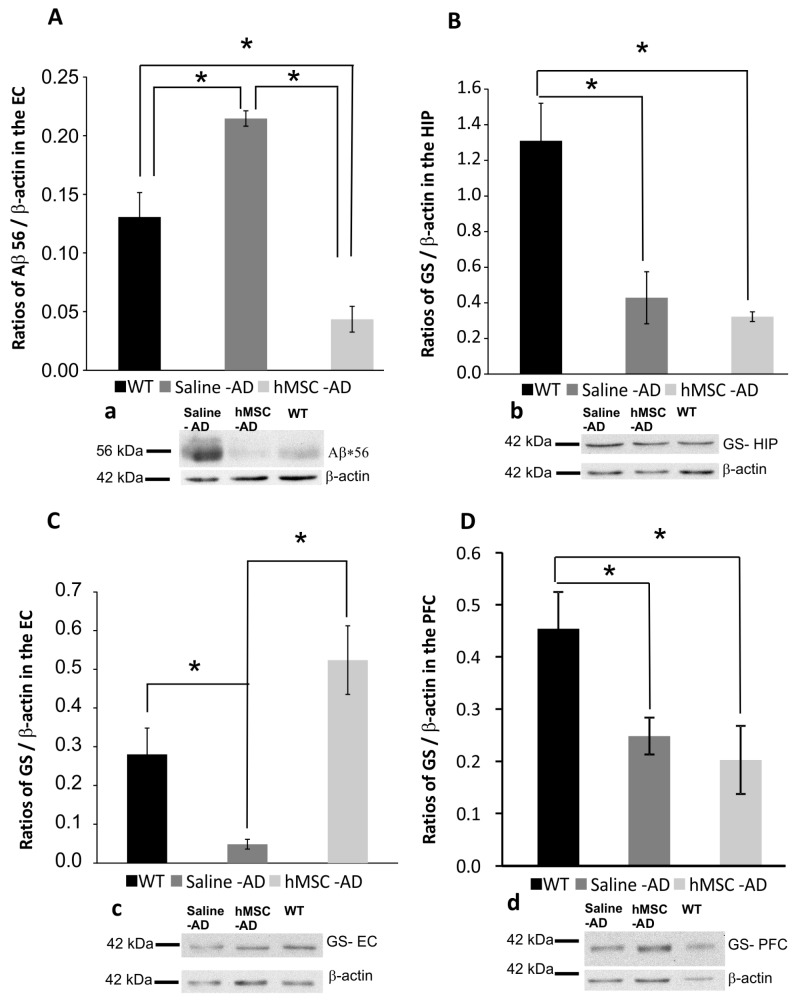
Western blotting demonstrated that the levels of Aβ*56 were reduced in the entorhinal cortex after hMSC transplantation (**A**); The levels of glutamine synthase (GS) in the hippocampus (**B**) and prefrontal cortex (**D**) were significantly reduced compared to WT controls in both hMSC-treated and saline-treated 3xTg-AD mice. In contrast, the entorhinal cortex showed preserved levels of GS in hMSC-treated mice compared to saline-treated animals (**C**). Western blots are shown in **a**, **b**, **c**, and **d**, respectively. * *p* < 0.05. HIP: hippocampus; PFC: Prefrontal cortex; EC: Entorhinal cortex.

Amyloid plaques are one of the major histopathological hallmarks of AD. Nevertheless, the severity of cognitive decline and synaptic malfunction are not correlated with the prevalence of amyloid plaques in the brain. Recent studies [2,28,29,30,31] have shown that soluble Aβ, such as oligomers, correlated with the brain dysfunction of AD better than insoluble Aβ, thus leading to the proposal of the “oligomer hypothesis”. This hypothesis is based on studies of how small-sized Aβ oligomers affect plasticity and synaptogenesis, leading to the memory deficits and pathogenesis observed in animal models and humans with AD [32]. Studies in rodent models as well as in human patients revealed that Aβ*56 promoted the impairment of synaptic plasticity and cognition irrespective of neuronal loss or plaque burden [29,30,31,33,34,35]. We found unchanged levels of Aβ*56 in the prefrontal cortex and the hippocampus in all experimental groups. However, in the entorhinal cortex of MSC-injected 3xTg-AD mice, we observed decreased levels of potentially toxic Aβ*56 compared to the saline-injected group (saline *vs.* MSC, *p* < 0.01). Conversely, the levels of Aβ*56 in the saline-injected 3xTg-AD group were significantly higher than in both WT and MSC-treated Tg mice (saline *vs.* WT, *p* < 0.01; saline *vs.* MSC, *p* < 0.001) (Figure 2A).

Astrocytes are principal protectors against excitotoxicity and are key elements in glutamate homeostasis and metabolism. Glutamate taken up by astrocytes (by the glutamate transporters Glutamate aspartate transporter (GLAST) and Glutamate transporter-1 (GLT-1) is converted into glutamine by glutamine synthetase (GS) and then transported back to neurons, where it is used for conversion into glutamate and γAminobutiric acid (GABA). The downregulation of GS and glutamate transporters has been reported in many neurological disorders and diseases, including AD [36,37,38,39,40,41,42,43]. We investigated the influence of transplanted hMSCs on the expression of GS, GLT-1 and GLAST in specific brain regions: the hippocampus, the entorhinal cortex and the prefrontal cortex. We found a significant decrease of GS levels between 3xTg-AD animals (both the saline-treated and MSC-grafted groups) and the non-transgenic controls in the hippocampus and prefrontal cortex (MSC *vs.* WT, *p* < 0.05; saline *vs.* WT, *p* < 0.05) (Figure 2B,D). There was no statistical difference in GS levels between the saline-treated and MSC-transplanted groups (*p* > 0.05). In contrast, the levels of GS in the entorhinal cortex significantly decreased in the saline-injected group compared to the non-transgenic WT group (*p* < 0.05) (Figure 2C). However, MSC application helped to preserve the levels of GS in the entorhinal cortex, where the GS levels remained significantly higher than those found in the saline-treated animals (*p* < 0.01) (Figure 2C). Considering GLT-1 and GLAST levels, no significant differences were observed between any of the groups.

### 2.3. Neurogenesis

**Figure 3:**
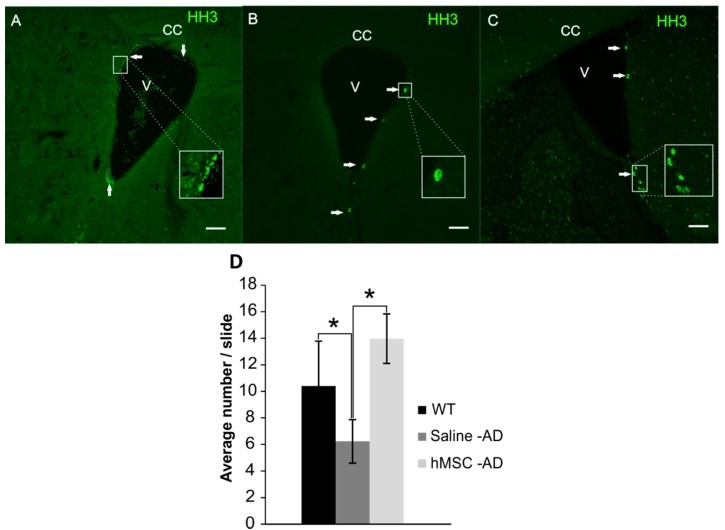
Neurogenesis in the subventricular zone (SVZ) in WT control animals (**A**); saline-treated 3xTg-AD mice (**B**); and MSC-treated 3xTg-AD mice (**C**). White arrows show histone H3 (HH3)-positive cells. Scale bar 50 μm (**A**–**C**). The bar graph shows the mean number of HH3-positive cells/slice in the SVZ area (**D**). * *p* < 0.05. “CC”—Corpus Calosum; “V”—Ventricle.

We analysed the effect of hMSCs on neurogenesis, which is highly impaired in AD. Cell proliferation was visualized using antibodies against phosphorylated phospho-histone H3 (HH3). We observed increased proliferative capabilities in the subventricular zone (SVZ) of MSC-treated 3xTg-AD mice six months after transplantation (Figure 3A), where we found clusters of cells positive for HH3, similarly as in age-matched WT controls (Figure 3C). In contrast, only a few HH3-positive cells were found in the saline-treated group (Figure 3B). Statistical analysis revealed significant differences at 14 months of age between WT mice and saline-treated animals (WT *vs.* saline; *p* < 0.05), while MSC-treated 3xTg-AD mice showed increased neurogenesis in the SVZ when compared to saline-treated animals (MSC *vs.* saline *p* < 0.05) (Figure 3D). None of the grafted hMSCs were detected six months after transplantation.

## 3. Discussion

In the present study, we examined the effect of an intraventricular implantation of hMSCs on spatial reference memory and working memory deficits in 3xTg-AD mice at six months post-transplantation. The significant reduction of Aβ*56 and the upregulation of GS levels in the entorhinal cortex may underpin the protective effects of MSC treatment on working memory loss. MSCs have been extensively used in experimental treatments of AD and have shown their potential in attenuating memory loss. Growing evidence supports the beneficial effects of stem cell implantation on AD and facilitates our understanding of AD pathogenesis and the creation of adequate AD models *in vitro* as well as *in vivo*. In primary cultured hippocampal neurons, amyloid β-induced apoptotic cell death was decreased by co-cultivation with MSCs due to the activation of the cell survival signaling pathway [10]. However, this anti-apoptotic effect of MSCs is hard to observe *in vivo* in animal models since the triple transgenic mouse model of AD does not display neural loss [44,45]. The alleviation of AD symptoms following MSC implantation has been described in several different animal models of AD, such as the APP/PS1, PSAPP, and Tg2576 mouse models. These beneficial effects of MSCs reportedly have included the improvement of spatial memory impairment, a reduction of Aβ plaques as well as soluble Aβ, modulation of the inflammatory response and increased synaptogenesis [10,46,47,48,49].

The MWM test is known to assess spatial memory [50] in which the hippocampus and entorhinal cortex play important roles [51,52]. In our 3xTg-AD mice, there was no significant difference in reference memory between five-month-old and 14-month-old animals (data not shown) nor between saline- and hMSC-treated mice. The reason why reference memory was not impaired in the 3xTg-AD animals is most likely related to the lesser development of pathological changes, particularly to the limited plaque formation seen in these mice. Therefore, we extended our investigation to include working memory, which is impaired in the early stage of AD [53]. We observed that hMSC treatment prevented the development of a working memory deficit in the treated animals. The 3xTg-AD animals used in our experiments displayed plaque formation and Aβ accumulation later than at 14 months of age, which does not correspond to the original model description [25]. We observed only intracellular Aβ deposits and the beginning of plaque formation at 14 months of age; these were fully developed at 20 months of age (Figure S1). On the other hand, we detected fewer intracellular deposits and a delayed start of plaque formation in animals treated with MSCs. However, we cannot prove that these findings are solely the result of the stem cell treatment (Figure S1). Although a therapeutic effect of MSCs related to the reduction of Aβ deposits was not proven in our study because of the absence of a clear pathological appearance of Aβ, we observed a decrease in the level of Aβ*56 oligomers in the entorhinal cortex, which is involved in spatial as well as working memory formation and is subject to pathological changes in 3xTg-AD mice [54]. Yeh and colleagues described a significant reduction in the surface area and volume of Glial fibrillary acidic protein (GFAP)-positive cells in the entorhinal cortex of 3xTg-AD mice at very early ages (one month) compared to WT controls. This reduction was not accompanied by a decrease in the numerical density of GS-immunoreactive cells or by a decrease in GS content (measured by optical density), since both parameters remained constant between one and 12 months of age [55,56]. We found decreased levels of GS (by Western blot) in the hippocampus, prefrontal cortex and entorhinal cortex of saline-treated 3xTg-AD mice at 14 months of age, and this downregulation in the entorhinal cortex was prevented by MSC treatment. We speculate that MSCs affect the protection of astrocytes rather than their proliferation, since no increased HH3 positivity was observed in the entorhinal cortex (EC) of MSC-treated animals. To confirm this, it would be necessary to perform further analyses and double staining for HH3/GFAP at different time points. A similar decrease in GS levels was reported by us in the hippocampus at 9–18 months of age and in the prefrontal cortex at 6–12 months of age in the same animal model [57,58].

Neurogenesis in 3xTg-AD animals is impaired in both neurogenic areas, the SVZ and the hippocampus [59,60]. We observed increased proliferation in the SVZ of MSC-treated animals. We speculate that these cells can be of neuronal origin, since in previous studies, less than 1%–2% of HH3-positive cells expressed the glial marker GFAP [59,60]. To determine the detailed phenotype of the newborn cells, further analysis would be required. We also determined the number of HH3-positive cells in the hippocampus; however, we did not find any differences between the animal groups. This correlates with the results of our previous study, in which neurogenesis in the hippocampus declined in aged WT mice as well as in 3xTg-AD animals [59].

Animal models exhibiting the pathological hallmarks of AD do not display neuronal loss. Therefore, we were not able to assess the effect of MSCs on the number of dying and lost neurons. Nevertheless, in several other pathologies MSCs act mostly via the production of growth factors and/or the reduction of inflammation [61,62]. Therefore the route and timing of cell application are important factors in promoting their effect on the host tissue. In our study, we applied cells at the beginning of AD onset (at eight months of age) in a single intraventricular injection. The effect of cell therapy was observed six months later, at the age of 14 months. We did not find any grafted cells homing into the brain parenchyma, therefore it is most likely that the cells exert their effect only temporarily. In the study of Lee [10], MSCs were grafted repeatedly into both hippocampi of APP/PS1 Tg mice for a period of one month, and the animals were sacrificed one month later. The animals were tested for spatial memory only three days after the last cell application. This experimental set up revealed an immediate and more robust short-term effect of MSCs; however, in our study, we were able to detect long-term changes even six months after cell application. It is likely that repeated application might lead to an even more pronounced effect of the cell therapy.

The intrahippocampal application of stem cells is used in order to target the hippocampus more directly so as to improve cognitive functions and neurogenesis in the dentate gyrus and to prevent AD pathogenesis. Intraventricular application, on the other hand, enables the implanted cells to more easily exert a paracrine effect on the adjacent areas, as seen in the levels of GS and Aβ*56 oligomers in the enthorhinal cortex. In addition, we observed increased neurogenesis in the SVZ in the hMSC-treated animals, which might play an important role in protecting against neuronal loss in the AD brain. To unravel the mechanism(s) underlining the effect of MSCs, further studies are required. These should include analysis at different time points after MSC application (one week, one month, three months) to clarify the survival of MSCs, the effect of MSCs on neuroinflammation by means of cytokine analysis and microglia detection, and the effect of MSC application at the late stage of the disease (grafting at 12 or 14 months). Finally, different routes of application should be compared.

## 4. Material and Methods

### 4.1. Animals and Cell Transplantation

The 3xTg-AD mouse strain (LaFerla, Irvine, CA, USA), harboring three transgenes of PS1 (M146V), tau (P301L) and APP (SWE), was used. The 3xTg-AD strain is generated from a hybrid of C57BL/6 mice and F1 of 129X1/SvJ and 129S1/Sv. The transgenic mice were injected with hMSCs (*n* = 16) or 2 µL of saline (*n* = 14) into the left lateral ventricle at 8 months of age. In parallel with the treated transgenic mice, non-treated non-transgenic mice (C57BL6 strain, *n* = 14) were used as age-matched wild-type (WT) controls. All experiments were performed in accordance with the European Communities Council Directive of 22 September 2010 (2010/63/EU) regarding the use of animals in research and were approved by the Ethics Committee of the Institute of Experimental Medicine ASCR, Prague, Czech Republic.

### 4.2. Human MSC Cultures

Human MSCs were prepared under Good manufacturing practice (GMP) conditions and supplied as a 1.5 mL cell suspension in Nunc tubes by Bioinova Ltd., Prague, Czech Republic, under the product name “Suspension of Autologous MSC 3P”. The mononuclear fraction containing MSCs was separated from the bone marrow by gradient centrifugation using 25% Gelofusine (B. Braun, Melsungen, Germany). The cells were expanded in media containing α Minimum Essential Medium (MEM) Eagle without deoxyribonucleotides, ribonucleotides and UltraGlutamin (Lonza, Basel, Switzerland) supplemented with 5% mixed allogeneic thrombocyte lysate (BioInova, Prague, Czech Republic) and 10 μg/mL gentamicin (Lek Pharmaceuticals, Ljublanja, Slovenia). Cells from the second passage were analyzed and used for transplantation. The expression of specific surface markers was assessed using fluorescent-activated cell sorting (FACS) analysis (FACSAria flow cytometer, BD Biosciences, San Diego, CA, USA). The cells were positive for CD105, CD73 and CD90 and negative for CD45, CD34, CD14 or CD11b, CD79α and HLA-DR (Human Leukocyte Antigens locus DR).

### 4.3. Transplantation

At 8 months of age, 3xTg-AD mice were injected with human MSCs (6 × 10^4^ cells/2 µL) or 2 µL of saline into the left lateral ventricle. Animals were anesthetized by an injection of ketamine (40 mg/kg) and xylazine (4 mg/kg), and anesthesia was maintained during the surgical procedure by 0.8% isoflurane in air at a flow rate of 50–60 mL/min. The animal’s head was secured in a stereotaxic device (Stoelting Co., Wood Dale, IL, USA). An incision was made on the scalp to expose the surface of the skull. A small hole was drilled in the skull over the left lateral ventricle (coordinates from bregma: anteroposterior = 0 mm, mediolateraly = 1 mm, dorsoventraly = 2 mm). 2 µL of cell suspension were injected through a glass pipette at a rate of 1 µL/minute using a Nano-Injector (Stoelting Co., Wood Dale, IL, USA). The glass pipette was kept in place after injection for a further 5 min. to prevent leakage of the cell suspension. The saline treated transgenic controls received 2 µL of saline. Triple-drug immunosuppression was used to prolong the survival of the transplanted cells Cyclosporine (10 mg/kg), azathioprine sodium (4 mg/kg, Imuran, glaxosmithkline pharmaceuticals s.a., Poznaň, Poland), and ampicillin (50 mg/kg) were administered one day before cell implantation and then every day throughout the experiment (for 6 months). Methylprednisolon (Sol-Medrol) was applied at a dose of 2 mg/kg, tapered gradually, and discontinued by three months after transplantation.

### 4.4. Behavioral Evaluation

#### 4.4.1. Spatial Reference Memory

Spatial reference memory was evaluated using a conventional Morris water maze (MWM) test. Mice (saline-injected 3xTg-AD, *n* = 14; MSC-injected 3xTg-AD, *n* = 16; and WT controls without treatment, *n* = 14) were trained in a circular pool (1.2 m in diameter) filled with water made opaque by a nontoxic white coloring agent at a constant temperature (25 °C). An escape platform (10 cm in diameter) was positioned in one of the quadrants 1cm below the surface of the water. Mice were trained for 10 days and were given 4 trials per day, each of which was started from a different location within each of the quadrants. A probe trial, in which the hidden platform was removed from the pool, was performed 24 h after the last acquisition trial. The animals were trained to find the submerged platform within one minute using two fixed cues placed on the inner wall of the pool. Distal cues were avoided by surrounding the pool with a dark curtain. The latency to find the hidden platform, swimming distance, swimming velocity and visit frequency were automatically monitored with a video tracking system (VideoMot2, TSE systems GmbH, Bad Homburg, Germany).

#### 4.4.2. Working Memory

Spatial working memory (WM) was evaluated at 14 months of age using the MWM (WM-MWM). The test, in which the animal had to find a different location of the hidden platform within 2 min during four consecutive trials with a 15 s inter-trial interval, was administered for five days. The submerged platform was randomly placed in one of the four quadrants every day, while the same starting position was used for all four trials on each day. The platform also remained fixed in the same location during the four successive daily trials. The wall of the tank was decorated with 4 different visual cues (different shape and color). The first and second trials each day were used to evaluate WM by measuring the difference in time between the two trials and calculating what percentage of the time in s was saved in finding the platform during the second trial. The third and fourth trials were used to train the animal to learn the position for the day.

### 4.5. Western Blotting

At the end of a series of behavioral experiments, the mice (*n* = 8 for each group) were deeply anesthetized with ketamine (100 mg/kg) and xylazine (20 mg/kg) and decapitated. The brain was removed from the skull, and samples of different brain regions—the prefrontal cortex (PFC), entorhinal cortex (EC) and hippocampus (HIP)—were collected by dissecting on ice. The samples were then homogenized in RIPA buffer containing protease inhibitors, and the tubes were centrifuged at 1000 rpm for 5 min. The lysate was placed into new tubes for western blot measurements and stored in a freezer at −80 °C. SDS PAGE electrophoresis with 12% Tris-Glycing gel was used, and a protein ladder (Life Technologies, Carlsbad, CA, USA) was included to verify the proper size of the protein of interest. Proteins were transferred onto a nitrocellulose membrane (Whatman paper, Bio-Rad, Hercules, CA, USA) for further manipulation. Mouse primary antibodies against Glutamine Synthase (GS) (Millipore, Darmstadt, Germany), Glutamate Transporter-1/Excitatory Amino Acid Transporter-2 (GLT-1/EAAT2, Cell Signaling Technology, Danvers, MA, USA), Glutamate Aspartate Transporter/Excitatory Amino Acid Transporter-1 (GLAST/EAAT1 Cell Signaling Technology, Danvers, MA, USA), amyloid β (Covance, Princeton, NJ, USA) and β-actin (Sigma-Aldrich, Munich, Germany) were used to probe the membrane, followed by secondary antibodies (goat anti-mouse) conjugated with horseradish peroxidase to visualize primary antibody reactivity (Jackson ImmunoReserach, West Grove, PA, USA). To evaluate the strength of the signal, enhanced chemiluminescence (ECL) solutionwas used to transfer the signal on X-ray films using development and photography fixer bath. ECL solution was mixed 1:1 from Solutions 1 and 2; Solution 1 contains luminol and cumaric acid (both Sigma-Aldrich) in DMSO, while solution 2 is H_2_O_2_ (Merk, Branchburg, NJ, USA). Both solutions were dissolved in TRIS (pH 8.5). The signal bands were evaluated using ImageJ software (developed by NIH, Bethesda, MD, USA), and graphs were created showing the arithmetic means of each group (Microsoft Excel 2010, Microsoft, Redmond, WA, USA).

### 4.6. Immunohistochemical Staining and Microscopy

After finishing the behavioral experiments (at 14 months of age), all three groups of animals (*n* = 6 for WT controls and saline treated Tg; *n* = 8 for the MSC-treated group) were sacrificed under deep anesthesia and intracardialy perfused with 4% paraformaldehyde solution. After perfusion, the brains were removed and post-fixed in the same fixative solution overnight. Subsequently, the brains were incubated in 20% sucrose at 4 °C until equilibrated. Cryosections were made using a Cryocut (Leica, Wetzlar, Germany) to obtain serial coronal sections (30 µm thick) and stored at −20 °C until stained. Primary antibodies against histone H3 (HH3) and human nuclei (HuNu) (both Millipore, Darmstadt, Germany) were used to detect proliferating cells and cells of human origin, respectively. HH3 antibody (Anti-phospho-Histone H3 (Ser10) Antibody) is a mitosis marker for the detection of Histone H3 phosphorylated at serine 10. Immunostaining with phospho-specific antibodies in mammalian cells reveals mitotic phosphorylation of H3 Thr3 in prophase and its dephosphorylation during anaphase. To determine the level of Aβ intracellular deposits and plague formation, an antibody against β amyloid, 6E10 (1:200, Covance), which reacts to abnormally processed isoforms as well as precursor forms of human β amyloid, was used. We detected weak cross-reactivity on tissue sections from WT animals. To visualize the primary antibodies, Alexa Fluor 488 and 594 conjugated goat anti-mouse IgG secondary antibodies (both Invitrogen, Carlsbad, CA, USA) were used. Antibody staining was detected and analyzed using fluorescence and confocal microscopes (AXIO Observer D1/LSM5 Duo, Carl Zeiss, Oberkochen, Germany). The number of HH3-positive cells was counted manually in the SVZ area of the WT controls and in the MSC- and saline-treated 3xTg animals.

### 4.7. Statistical Analysis

All values are shown as mean ± standard error of mean (SEM). One-way or two-way ANOVA was used to assess the statistical differences between groups as appropriate. ANOVA testing was followed by the Student-Neuman-Keuls *post hoc* test when necessary. Differences were considered statistically significant if *p* < 0.05.

## 5. Conclusions

Our study indicates that the transplantation of hMSCs partially prevents AD progression via the reduction of potentially neurotoxic Aβ*56 oligomers, an increase of endogenous neurogenesis in the SVZ, and the partial prevention of excitotoxicity effects by maintaining glutamate transport systems in the entorhinal cortex, thus leading to a smaller working memory deficit at 14 months of age. However, to further unravel the possible mechanisms underlying the beneficial effects of cell therapy, it will be necessary to examine 3xTg-AD mice at several time points after grafting, during disease progression up to 20 months of age, and to focus more closely on neuroinflammation.

## Supplementary Materials

Supplementary materials can be found at http://www.mdpi.com/1422-0067/17/2/152/s1.
