# Supplementary material for: Mesenchymal Stem Cells Preserve Working Memory in the 3xTg-AD Mouse Model of Alzheimer’s Disease
Source: Int J Mol Sci. 2016 Jan 25;17(2):152. doi: 10.3390/ijms17020152 (PMC4783886; doi:10.3390/ijms17020152)
Supplement: Supplementary file 1 [file ijms-17-00152-s001.pdf]

Supplementary Materials: Mesenchymal Stem Cells Attenuate Working Memory in the 3xTg-AD Mouse Model of Alzheimer's Disease

Jiri Ruzicka, Magdalena Kulijewicz-Nawrott, Jose Julio Rodrigez-Arellano, Pavla Jendelova and Eva Sykova

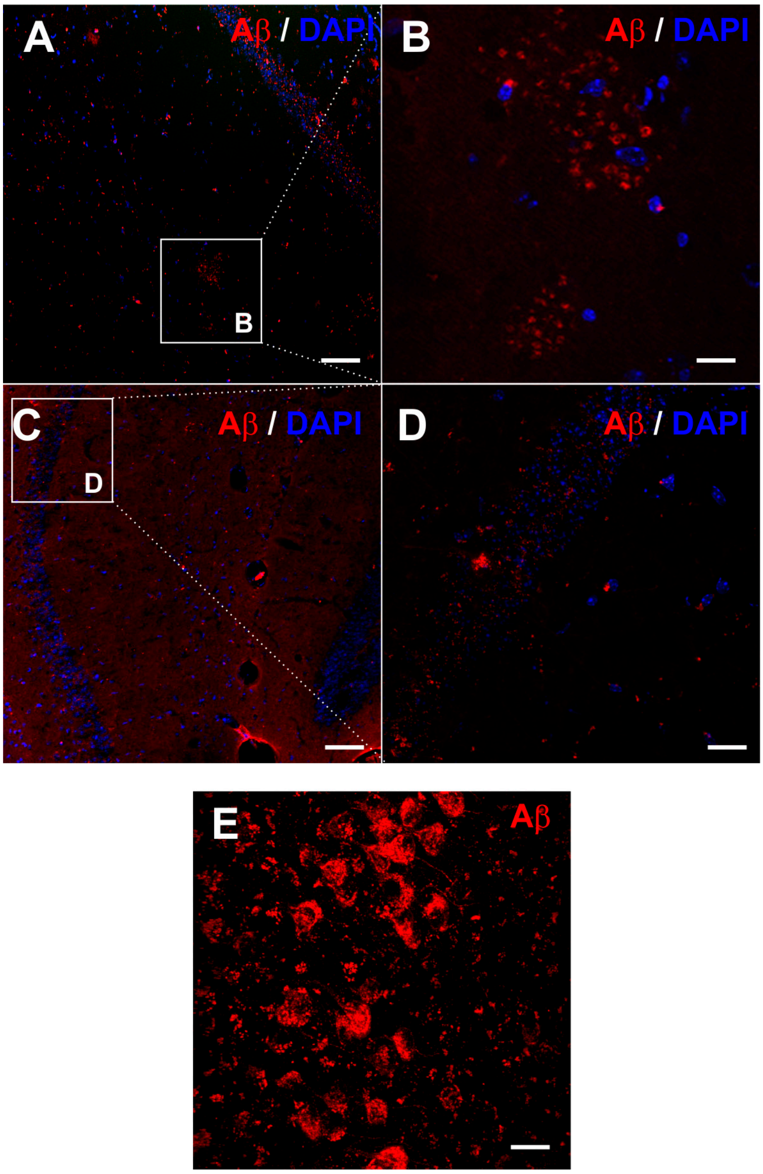

Figure S1. Aβ intracellular deposits and plague formation (red) at 14 months of age in the hippocampus of saline- (**A**, detail **B**) and hMSC-treated AD mice (**C**, detail **D**). Aβ intracellular deposits and plague formation (red) at 20 months of age in the hippocampus of a 3xTg AD mouse (**E**). Scale bars: 20 μm (**A**,**C**) and 60 μm (**B**,**D**,**E**). DAPI: 4′,6-Diamidine-2′-phenylindole dihydrochloride, blue.
